# Standardization of the Italian ALS-CBS™ Caregiver Behavioral Questionnaire

**DOI:** 10.3389/fpsyg.2022.1107001

**Published:** 2023-01-20

**Authors:** Edoardo Nicolò Aiello, Federica Solca, Lucia Catherine Greco, Antonino La Tona, Silvia Torre, Laura Carelli, Claudia Morelli, Alberto Doretti, Eleonora Colombo, Stefano Messina, Debora Pain, Alice Radici, Andrea Lizio, Jacopo Casiraghi, Federica Cerri, Agostino Brugnera, Angelo Compare, Susan Woolley, Jennifer Murphy, Lucio Tremolizzo, Ildebrando Appollonio, Federico Verde, Valeria Ada Sansone, Christian Lunetta, Vincenzo Silani, Nicola Ticozzi, Barbara Poletti

**Affiliations:** ^1^Department of Neurology and Laboratory of Neuroscience, IRCCS Istituto Auxologico Italiano, Milan, Italy; ^2^Ph.D. Program in Neuroscience, School of Medicine and Surgery, University of Milano-Bicocca, Milan, Italy; ^3^Neuromuscular Omnicentre (NEMO), Fondazione Serena Onlus, Milan, Italy; ^4^NeMO Lab, ASST Grande Ospedale Metropolitano Niguarda, Milan, Italy; ^5^Department of Human and Social Sciences, University of Bergamo, Bergamo, Italy; ^6^Istituti Clinici Scientifici Maugeri IRCCS, Department of Neurorehabilitation of Milan Institute, Milan, Italy; ^7^Syneos Health, Morrisville, NC, United States; ^8^Biogen Inc., Cambridge, MA, United States; ^9^School of Medicine and Surgery, University of Milano-Bicocca, Milan, Italy; ^10^Department of Pathophysiology and Transplantation, “Dino Ferrari” Center, Università degli Studi di Milano, Milan, Italy; ^11^Department of Biomedical Sciences of Health, University of Milan, Milan, Italy

**Keywords:** ALS Cognitive Behavioral Screen, amyotrophic lateral sclerosis, behavior, frontotemporal degeneration, dysexecutive

## Abstract

**Background:**

The present investigation aimed at testing the psychometrics and diagnostics of the Italian version of the Caregiver Behavioral Questionnaire (CBQ) from the ALS Cognitive Behavioral Screen (ALS-CBS™), as well as its case–control discrimination, in a cohort of non-demented patients with ALS.

**Methods:**

The caregivers of *N* = 265 non-demented patients with ALS and *N* = 99 healthy controls (HCs) were administered the CBQ and the Edinburgh Cognitive and Behavioural ALS Screen-Carer Interview (ECAS-CI). For *N* = 98 patients, an in-depth behavioural/psychopathological assessment *via* the Frontal Behavioural Inventory (FBI), the Dimensional Apathy Scale (DAS), the State and Trait Anxiety Inventory-Form Y (STAI-Y), and the Beck Depression Inventory (BDI) was also available. Factorial and construct validity, internal reliability, and diagnostics against an abnormal ECAS-CI score were tested in patients. Case–control discrimination was explored through logistic regression.

**Results:**

The CBQ was internally reliable (McDonald’s ω = 0.90) and underpinned by a simple, unidimensional structure; it converged with ECAS-CI, FBI, and DAS scores and diverged from STAI-Y and BDI ones. A cutoff of ≤ 33 accurately detected abnormal ECAS-CI scores (AUC = 0.85), yielding optimal error- and information-based diagnostics. The CBQ was independent of demographic and disease-related variables and discriminated patients from HCs (*p* < 0.001).

**Discussion:**

The Italian version of the CBQ from the ALS-CBS™ is a valid, reliable, diagnostically sound, and feasible screener for detecting frontotemporal-like behavioural changes in non-demented patients with ALS. Its adoption is thus recommended within clinical practice and research in the view of providing preliminary information on whether the administration of more extensive behavioural instruments is needed.

## 1. Background

Up to 50% of amyotrophic lateral sclerosis (ALS) patients without dementia show frontotemporal-like changes in behavior over the course of the disease (Strong et al., 2017; Feldman et al., 2022). Since such behavioral dysfunctions negatively affect their prognosis and care management (Huynh et al., 2020), their early detection *via* clinimetrically sound, disease-specific screeners are clinically pivotal in this population (Gray and Abrahams, 2022). In addition, behavioral measures have been addressed within the context of both observational and interventional studies addressing patients with ALS (Beswick et al., 2021).

Among ALS-specific behavioral scales, the proxy-report checklist included within the ALS Cognitive Behavioral Screen (ALS-CBS™)—that is, the Caregiver Behavioral Questionnaire (CBQ) (Woolley et al., 2010)—has been shown to be cross-sectionally/longitudinally feasible and featured by optimal clinimetrics, at least as far as its original, English version is concerned (Simon and Goldstein, 2019; Gosselt et al., 2020).

In Italy, a back-translated version of the CBQ is available, with its convergent validity having been tested within the context of the Italian standardization of the ALS-CBS™ (Tremolizzo et al., 2020). In addition, evidence supportive of its cross-sectional feasibility for detecting behavioral changes in Italian, non-demented patients with ALS has been recently delivered (Greco et al., 2022).

Nevertheless, to this day, no full psychometric, diagnostic, and feasibility studies have been performed in Italy on the CBQ. Hence, the present investigation aimed at delivering, in a retrospective cohort of Italian, non-demented patients with ALS, (1) evidence on its factorial and construct validity, (2) internal reliability, (3) diagnostic properties, and (4) an assessment of its capability to discriminate them from healthy controls (HCs).

## 2. Materials and methods

### 2.1. Participants

Data on *N* = 265 patients with ALS (Brooks et al., 2000) referred to three Northern Italian centers (i.e., IRCCS Istituto Auxologico Italiano; NEMO, Fondazione Serena Onlus; and Istituti Clinici Scientifici Maugeri IRCCS) between 2020 and 2022 was retrospectively retrieved. In addition, *N* = 99 HCs were prospectively recruited at the IRCCS Istituto Auxologico Italiano by means of authors’ personal acquaintances and advertising. Neither patients nor HCs had (other) neurological/psychiatric disorders or general-medical conditions possibly affecting neuropsychological functions (i.e., unstable internal medical/metabolic diseases or system/organ failures). No patient met either Gorno-Tempini et al. (2011) and Rascovsky et al. (2011) criteria for FTD. This study was approved by the local ethics committees; participants provided informed consent, and data were treated according to current regulations.

### 2.2. Materials

#### 2.2.1. Primary measures

Patients’ and HCs’ first-degree relatives or spouses/partners were administered the Italian versions of the CBQ (Tremolizzo et al., 2020) and Edinburgh Cognitive and Behavioural ALS Screen-Carer Interview (ECAS-CI) (Poletti et al., 2016, 2022). The ECAS-CI was addressed as the main comparator against which the construct validity of the CBQ was tested (Sect. 2.3.1).

The CBQ is a 15-item, caregiver-report checklist covering the key, FTD-like behavioral signs/symptoms—that is, dysexecutive features of either an apathetic or a disinhibited nature—with items 6 and 15 instead targeting attention and language deficits, respectively. Each Likert-scaled item inquires about changes that occurred after the onset of the disease, ranging from 0 (“large change”) to 3 (“no change”); the total score on the CBQ, thus, ranges from 0 to 45, with lower scores indexing a higher level of behavioral dysfunction. The Italian CBQ is available upon the reasonable request of interested practitioners/researchers to the corresponding author. The ECAS-CI is likewise a caregiver-report checklist, comprising 13 dichotomous items (i.e., requiring “yes-or-no” answers) falling into the following clusters: disinhibition, apathy, loss of sympathy/empathy, perseveration, altered eating habits, and psychosis. The total score on the ECAS-CI ranges from 0 to 13, with higher scores on the ECAS-CI indexing a higher level of behavioral dysfunction. Both the CBQ and the ECAS-CI take no longer than 5 min to be completed.

#### 2.2.2. Secondary measures

All patients had been screened for cognitive impairment by the cognitive sections of the Italian ALS-CBS™ (Tremolizzo et al., 2020) and ECAS (Poletti et al., 2016) and assessed for motor-functional outcome *via* the ALS Functional Rating Scale-Revised (ALSFRS-R) (Cedarbaum et al., 1999).

In addition, out of the whole cohort, *N* = 98 patients had undergone an in-depth behavioral and psychopathological assessment *via* the Frontal Behavioural Inventory (FBI) (Alberici et al., 2007), Dimensional Apathy Scale (DAS) (Santangelo et al., 2017), State and Trait Anxiety Inventory-Form Y (STAI-Y1 and STAI-Y2—for the state and trait anxiety, respectively) (Spielberger et al., 1971), and Beck Depression Inventory (BDI) (Beck et al., 1961). The scores on such scales were addressed to further test the construct validity of the CBQ (Sect. 2.3.1).

### 2.3. Statistics

Analyses were performed using R 4.1^1^ and jamovi 2.3 software.^2^ The significance level was set at α = 0.05.

#### 2.3.1. Psychometrics

In patients, internal reliability and factorial validity of the CBQ were tested by McDonald’s ω and principal component analysis (PCA), respectively. Construct validity of the CBQ was assessed against ECAS-CI, FBI, DAS, STAI-Y1/-Y2, and BDI scores by means of Bonferroni-corrected Spearman’s correlations—because the vast majority of such measures were not distributed normally (i.e., skewness and kurtosis values >|1| and |3|, respectively) (Kim, 2013). The minimum sample sizes for internal reliability and construct validity analyses were set at *N* = 20 and *N* = 80, respectively, in agreement with Hobart et al.’s (2012) recommendations—which are specific to the standardization of psychometric scales within clinical neurological research. As far as the sample size for the PCA, Kyriazos’ (2018) empirical rule was referred to, according to which *N* = 100 observations are sufficient to run such an analysis.

#### 2.3.2. Diagnostics

In patients, the diagnostic properties of the CBQ—that is, sensitivity, specificity, positive and negative predictive values (PPV; NPV), and positive and negative likelihood ratios (LR +; LR–)—were computed, through a receiver-operating characteristics (ROC) analysis, at the optimal cutoff identified *via* Youden’s *J* statistic. An above-cutoff ECAS-CI score (Poletti et al., 2022) was addressed as the positive state (i.e., the presence of behavioral changes). In ROC analysis, the minimum sample size was set at *N* = 82, by forecasting a prevalence of up to 50% of patients with an above-cutoff ECAS-CI (i.e., allocation ratio of 1; 41 patients with an above- *vs.* below-cutoff ECAS-CI) (Strong et al., 2017), an AUC = 0.7, α = 0.05, and 1–β = 0.95 (Goksuluk et al., 2016).

#### 2.3.3. Case–control discrimination

Case–control discrimination of the CBQ was tested through a logistic regression (LR) that regarded the group as the dichotomous outcome (i.e., patients *vs.* HCs). Within such a model, age, education, and sex were entered as covariates—since the two groups were not matched for such variables [age: *t*(361) = 8.67; *p* < 0.001; education: *t*(361) = –3.70; *p* < 0.001; sex: χ^2^(1) = 7.68; *p* = 0.006].

#### 2.3.4. Effect of demographic and disease-related confounders

In order to test whether demographic (i.e., age, education, and sex) or disease-related confounders (i.e., disease duration, ALSFRS-R scores, and presence of *C9orf72* hexanucleotide repeat expansion) affected CBQ scores, a negative binomial regression (NBR) (Aiello et al., 2020) was run by covarying for cognition (i.e., ECAS scores) and psychopathological features (i.e., STAI-Y1, STAI-Y2, and BDI scores). A reversed CBQ (computed as 45-CBQ scores) was addressed as the outcome within the NBR in order for the data to fit the underlying probability distribution. Indeed, the CBQ proved to be heavily left-skewed and overdispersed; by reversing its scale, a right-skewed, overdispersed empirical distribution yielded, which could be thereupon modeled by the negative binomial; this allowed the transformed variable to keep the original metric and thus operationalization of the outcome measure (Aiello et al., 2020). Such an expedient already proved to be effective in circumventing ceiling/floor effects and high inter-individual variability in behavioral scores (Iazzolino et al., 2022; Poletti et al., 2022).

## 3. Results

Table 1 shows the participants’ backgrounds and neuropsychological measures.

**TABLE 1:** Participants’ background and neuropsychological features.

	ALS	HCs	*p*
*N*	265	98	–
Age (years)	65.03 ± 10.9 (30–88)	55.84 ± 10.99 (24–88)	<0.001^a^
Sex (male/female)	52.1% / 47.9%	35.7% / 64.3%	0.006^b^
Education (years)	11.36 ± 4.2 (3–27)	13.18 ± 4.02 (5–22)	<0.001^a^
Disease duration (months)	38.71. ± 52.67 (1–328)	–	–
ALSFRS-R	32.38 ± 10.08 (4–48)	–	–
Genetics			
*C9orf72*/*SOD1*/*TARDBP*/ *FUS*	4.5% / 3.8% / 0.8% / 1.1%	–	–
ECAS-Total	99.67 ± 20.13 (19–134)	–	–
Impaired (%)	30.9%	–	–
ECAS-ALS-specific	73.71 ± 16.12 (14–98)	–	–
Impaired (%)	28.3%	–	–
ECAS-ALS-nonspecific	26.15 ± 5.46 (9–36)	–	–
Impaired (%)	21.5%	–	–
ECAS-CI	0.87 ± 1.34 (0–8)	0.02 ± .14 (0–1)	<0.001^c^
Impaired (%)	10.2%	–	–
ALS-CBS™–Cognitive section	14.64 ± 3.73 (3–20)	–	–
CBQ	38.68 ± 7.53 (4–45)	44.47 ± 1.45 (34–45)	<0.001^c^
CBQ-Anxiety (%)	37.9%	4.1%	<0.001^d^
CBQ-Depression (%)	35.6%	1%	<0.001^d^
CBQ-Fatigue (%)	64.8%	4.1%	<0.001^d^
CBQ-Lability (%)	27.2%	1%	<0.001^d^
FBI*	2.38 ± 3.07 (0–13)	–	–
DAS*	23.15 ± 9.70 (5–67)	–	–
STAIY-1*	54.92 ± 11.02 (33–81)	–	–
STAIY-2*	50.01 ± 10 (33–77)	–	–
BDI*	14.32 ± 9.49 (0–41)	–	–

ALS, amyotrophic lateral sclerosis; ALSFRS-R, Amyotrophic Lateral Sclerosis Functional Rating Scale-Revised; ALS-CBS, ALS Cognitive Behavioral Screen; ECAS, Edinburgh Cognitive and Behavioural ALS Screen; CI, Carer Interview; CBQ, Caregiver Behavioral Questionnaire; FBI, Frontal Behavioural Inventory; DAS, Dimensional Apathy Scale; STAIY, Stait- and Trait-Anxiety Inventory-Form Y; BDI, Beck Depression Inventory.

^a^*t*-statistics;

^b^χ^2^-statistics;

^c^Mann–Whitney *U*-statistics;

^d^Fisher’s exact test; and *available for *N* = 98 patients.

In patients, the CBQ proved to be underpinned by a simple, mono-component structure (43.2% of variance explained) with no interstitial items (loading *range* = 0.46–0.79), as well as to be internally reliable (McDonald’s ω = 0.90). At α_adjusted_ = 0.008, CBQ scores were strongly associated with the FBI [*r*_*s*_(98) = –0.80; *p* < 0.001] and ECAS-CI [*r*_*s*_(98) = –0.59; *p* < 0.001], moderately with the DAS [*r*_*s*_(98) = –0.34; *p* < 0.001], but not with STAI-Y1[*r*_*s*_(98) = –0.18; *p* = 0.0850], STAI-Y2 [*r*_*s*_(98) = –0.20; *p* = 0.052], and BDI scores [*r*_*s*_(98) = –0.21; *p* = 0.036].

**FIGURE 1:**
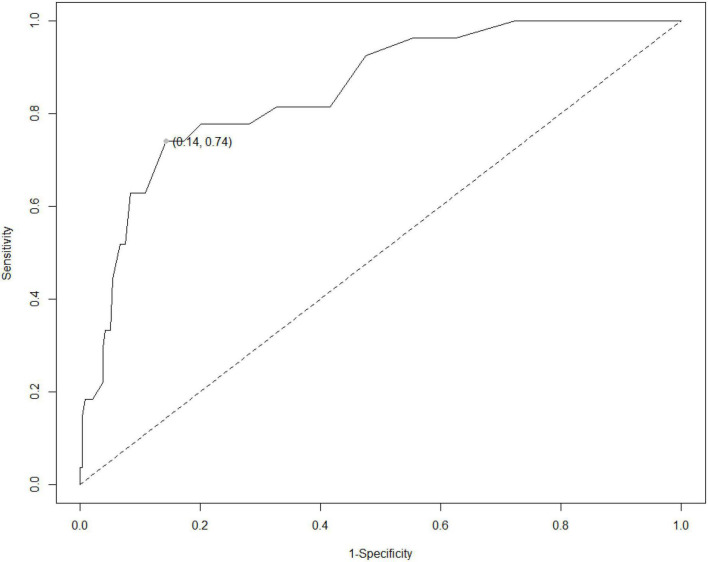
ROC curve for the CBQ against an above-cutoff value on the ECAS-CI. ROC, receiver-operating characteristics; ECAS-CI, Edinburgh Cognitive and Behavioural ALS Screen; CBQ, Caregiver Behavioral Questionnaire. The gray dot index the cutoff value identified *via* Youden’s index [≤ 33; *J* = 0.6; AUC = 0.85; *SE* = 0.04; 95% CI (0.78;0.92)]. The graphical representation was implemented by means of the *R* package *reportROC* (https://cran.r-project.org/web/packages/reportROC/reportROC.pdf).

Twenty-seven out of 265 patients presented with behavioral dysfunctions according to the ECAS-CI (10.2%). At the optimal cutoff of ≤ 33 (*J* = 0.6), the CBQ proved to be highly accurate [AUC = 0.85; *SE* = 0.04; 95% CI (0.78, 0.92)] (Figure 1), as well as to be featured by optimal error-based (sensitivity = 0.74; specificity = 0.86) and information-based diagnostics (PPV = 0.37; NPV = 0.97; LR + = 5.19; LR- = 0.30). A total of 20.4% of patients (54 out of 265) were classified as behaviorally dysfunctional by such a cutoff.

Net of ECAS, STAI-Y1, STAI-Y2, and BDI scores, the NBR revealed that no demographic or disease-related features affected CBQ scores in patients [χ^2^(1) ≤ 2.2; *p* ≥ 0.138].

Net of age, education, and sex, the LR revealed that CBQ scores effectively discriminated patients from HCs [*b* = –0.5; *p* < 0.001; OR = 0.61, 95% CI (0.51;0.73)] with a classification accuracy of 82.4%.

## 4. Discussion

The present study provides Italian practitioners and clinical researchers with the standardization of the CBQ from the ALS-CBS™ in non-demented patients with ALS. The CBQ proved to be (1) internally reliable, (2) underpinned by a unidimensional structure, (3) both convergently and divergently valid, (4) diagnostically sound, and (5) able to discriminate patients from HCs.

With regard to constructing validity, it is noteworthy that the CBQ selectively converged with dysexecutive behavioral features—as measured by the FBI, ECAS-CI, and DAS—by nonetheless diverging from psychopathological features—that is, STAI-Y1/STAI-Y2 and BDI scores. Moreover, the CBQ proved to be able to discriminate patients with ALS from HCs and to be independent of demographic and disease-related features. Interestingly, within the original, English study, similar findings have been detected: (1) convergent validity of the CBQ, (2) its independence of background confounders, and (3) its case–control discrimination (Woolley et al., 2010).

As far as the diagnostics is concerned, both error- and information-based properties proved to be optimal, with the exception of a poor PVV. However, it should be borne in mind that predictive values are prevalence-based metrics, with the PPV and NPV being directly and inversely related to the proportion of positive states within the study sample, respectively (Bossuyt, 2010). Therefore, given the low prevalence of the above-cutoff ECAS-CI scores, a low PPV was expected. By contrast, as not being based on disease prevalence, likelihood ratios are more generalizable and should be, thus, given more confidence by users when compared to predictive values themselves (Bossuyt, 2010).

Moreover, it is noteworthy that the cutoff herewith derived is relatively close to the original threshold proposed by Woolley et al. (2010) for differentiating non-demented patients with ALS without from those with behavioral involvement, that is, ≤ 36—which was featured, similarly to the results of the present investigation, by optimal diagnostics (accuracy = 86%; sensitivity = 0.82; specificity = 0.86; PPV = 0.82; NPV = 0.92).

This study is of course not exhaustive of all clinimetric and feasibility features that should be tested for a given behavioral screener (Aiello et al., 2022). First, evidence on test–retest and inter-rater reliability, as well as on criterion validity, is still lacking and, thus, needs to be addressed by future investigations. Moreover, within the present study, patients were not classified according to Strong et al. (2017) criteria: Hence, further studies are needed to test whether the CBQ is sensitive to the severity of behavioral dysfunction across the ALS-FTD *spectrum*—as defined according to the abovementioned nosographic system. Finally, it is advisable that the longitudinal feasibility of the CBQ be also tested—especially in light of the facts that the English version of the CBQ proved effective to track involutionary trends in behavior over time in patients with ALS (Woolley et al., 2018) and that behavioral functioning is acknowledged to worsen with disease progression (Consonni et al., 2021).

In conclusion, the CBQ is a valid, reliable, diagnostically sound, and feasible screener for behavioral dysfunctions in non-demented patients with ALS. Its adoption is, thus, recommended within clinical practice and research in the view of providing preliminary information on whether the administration of more extensive behavioral instruments currently available in Italy (Aiello et al., 2022) is needed.

## Data availability statement

The raw data supporting the conclusions of this article will be made available by the authors, without undue reservation.

## Ethics statement

The studies involving human participants were reviewed and approved by the Ethics Committee of IRCCS Istituto Auxologico Italiano (I.D.: 2013_06_25), by the Ethics Committee of Milano Niguarda Area 3 (I.D.: 393-09062021) and by the Ethics Committee of Istituti Clinici Scientifici Maugeri IRCCS (I.D.: CE 2495-12012021). The patients/participants provided their written informed consent to participate in this study.

## Author contributions

EA: conceptualization, analyses, drafting, and revision. FS and LG: data collection, drafting, and revision. ALT: analyses and revision. ST, LC, CM, AD, EC, SM, DP, AR, AL, JC, and FC: data collection and revision. AB, AC, SW, JM, LT, IA, FV, VAS, CL, VS, and NT: resources and revision. BP: conceptualization, drafting, revision and resources. All authors contributed to the article and approved the submitted version.

## Footnotes

1
https://cran.r-project.org/

2
https://www.jamovi.org/

## References

[B1] Aiello E. N. Depaoli E. G. Gallucci M. (2020). Usability of the negative binomial model for analyzing ceiling and highly-inter-individually-variable cognitive data. *Neurol. Sci.* 41 S273–S274.

[B2] Aiello E. N. D’Iorio A. Montemurro S. Maggi G. Giacobbe C. Bari V. (2022). Psychometrics, diagnostics and usability of Italian tools assessing behavioural and functional outcomes in neurological, geriatric and psychiatric disorders: a systematic review. *Neurol. Sci.* 43 6189–6214. 10.1007/s10072-022-06300-8 35932375 PMC9616758

[B3] Alberici A. Geroldi C. Cotelli M. Adorni A. Calabria M. Rossi G. (2007). The frontal behavioural inventory (Italian version) differentiates frontotemporal lobar degeneration variants from Alzheimer’s disease. *Neurol. Sci.* 28 80–86. 10.1007/s10072-007-0791-3 17464470

[B4] Beck A. T. Ward C. H. Mendelson M. Mock J. Erbaugh J. (1961). An inventory for measuring depression. *Arch. General Psychiatry* 4 561–571. 10.1001/archpsyc.1961.01710120031004 13688369

[B5] Beswick E. Park E. Wong C. Mehta A. R. Dakin R. Chandran S. (2021). A systematic review of neuropsychiatric and cognitive assessments used in clinical trials for amyotrophic lateral sclerosis. *J. Neurol.* 268 4510–4521. 10.1007/s00415-020-10203-z 32910255 PMC8563523

[B6] Bossuyt P. M. (2010). Clinical validity: defining biomarker performance. *Scand. J. Clin. Lab. Invest.* 70 46–52. 10.3109/00365513.2010.493383 20515277

[B7] Brooks B. R. Miller R. G. Swash M. Munsat T. L. (2000). El Escorial revisited: revised criteria for the diagnosis of amyotrophic lateral sclerosis. *Amyotrophic Lateral Sclerosis Motor Neuron Disord.* 1 293–299. 10.1080/146608200300079536 11464847

[B8] Cedarbaum J. M. Stambler N. Malta E. Fuller C. Hilt D. Thurmond B. (1999). The ALSFRS-R: a revised ALS functional rating scale that incorporates assessments of respiratory function. *J. Neurol. Sci.* 169 13–21. 10.1016/S0022-510X(99)00210-5 10540002

[B9] Consonni M. Dalla Bella E. Bersano E. Lauria G. (2021). Cognitive and behavioural impairment in amyotrophic lateral sclerosis: a landmark of the disease? a mini review of longitudinal studies. *Neurosci. Lett.* 754:135898. 10.1016/j.neulet.2021.135898 33862143

[B10] Feldman E. L. Goutman S. A. Petri S. Mazzini L. Savelieff M. G. Shaw P. J. (2022). Amyotrophic lateral sclerosis. *Lancet* 400 1363–1380. 10.1016/S0140-6736(22)01272-7 36116464 PMC10089700

[B11] Goksuluk D. Korkmaz S. Zararsiz G. Karaagaoglu A. E. (2016). easyROC: an interactive web-tool for ROC curve analysis using R language environment. *R. J.* 8 213–230. 10.32614/RJ-2016-042

[B12] Gorno-Tempini M. L. Hillis A. E. Weintraub S. Kertesz A. Mendez M. Cappa S. F. (2011). Classification of primary progressive aphasia and its variants. *Neurology* 76 1006–1014. 10.1212/WNL.0b013e31821103e6 21325651 PMC3059138

[B13] Gosselt I. K. Nijboer T. C. Van Es M. A. (2020). An overview of screening instruments for cognition and behavior in patients with ALS: selecting the appropriate tool for clinical practice. *Amyotrophic Lateral Sclerosis Frontotemporal Degenerat.* 21 324–336. 10.1080/21678421.2020.1732424 32157912

[B14] Gray D. Abrahams S. (2022). International evaluation of current practices in cognitive assessment for motor neurone disease. *Br. J. Neurosci. Nursing* 18 38–44. 10.12968/bjnn.2022.18.1.38

[B15] Greco L. C. Lizio A. Casiraghi J. Sansone V. A. Tremolizzo L. Riva N. (2022). A preliminary comparison between ECAS and ALS-CBS in classifying cognitive–behavioural phenotypes in a cohort of non-demented amyotrophic lateral sclerosis patients. *J. Neurol.* 269 1899–1904. 10.1007/s00415-021-10753-w 34410493

[B16] Hobart J. C. Cano S. J. Warner T. T. Thompson A. J. (2012). What sample sizes for reliability and validity studies in neurology? *J. Neurol.* 259 2681–2694. 10.1007/s00415-012-6570-y 22729386

[B17] Huynh W. Ahmed R. Mahoney C. J. Nguyen C. Tu S. Caga J. (2020). The impact of cognitive and behavioral impairment in amyotrophic lateral sclerosis. *Exp. Rev. Neurotherapeut.* 20 281–293. 10.1080/14737175.2020.1727740 32031423

[B18] Iazzolino B. Pain D. Laura P. Aiello E. N. Gallucci M. Radici A. (2022). Italian adaptation of the Beaumont Behavioral Inventory (BBI): psychometric properties and clinical usability. *Amyotrophic Lateral Sclerosis Frontotemporal Degenerat.* 23 81–86. 10.1080/21678421.2021.1946085 34279169

[B19] Kim H. Y. (2013). Statistical notes for clinical researchers: assessing normal distribution (2) using skewness and kurtosis. *Restorat. Dentistry Endodont.* 38 52–54. 10.5395/rde.2013.38.1.52 23495371 PMC3591587

[B20] Kyriazos T. A. (2018). Applied psychometrics: sample size and sample power considerations in factor analysis (EFA, CFA) and SEM in general. *Psychology* 9:2207. 10.4236/psych.2018.98126

[B21] Poletti B. Aiello E. N. Solca F. Torre S. Carelli L. Ferrucci R. (2022). Diagnostic properties of the Italian ECAS Carer Interview (ECAS-CI). *Neurol. Sci.* Online ahead of print. 10.1007/s10072-022-06505-x 36417015 PMC9925466

[B22] Poletti B. Solca F. Carelli L. Madotto F. Lafronza A. Faini A. (2016). The validation of the Italian Edinburgh cognitive and behavioural ALS screen (ECAS). *Amyotrophic Lateral Sclerosis Frontotemporal Degenerat.* 17 489–498. 10.1080/21678421.2016.1183679 27219526

[B23] Rascovsky K. Hodges J. R. Knopman D. Mendez M. F. Kramer J. H. Neuhaus J. (2011). Sensitivity of revised diagnostic criteria for the behavioural variant of frontotemporal dementia. *Brain* 134 2456–2477. 10.1093/brain/awr179 21810890 PMC3170532

[B24] Santangelo G. Raimo S. Siciliano M. D’Iorio A. Piscopo F. Cuoco S. (2017). Assessment of apathy independent of physical disability: validation of the dimensional apathy scale in Italian healthy sample. *Neurol. Sci.* 38 303–309. 10.1007/s10072-016-2766-8 27844173

[B25] Simon N. Goldstein L. H. (2019). Screening for cognitive and behavioral change in amyotrophic lateral sclerosis/motor neuron disease: a systematic review of validated screening methods. *Amyotrophic Lateral Sclerosis Frontotemporal Degenerat.* 20 1–11. 10.1080/21678421.2018.1530264 30585510

[B26] Spielberger C. D. Gonzalez-Reigosa F. Martinez-Urrutia A. Natalicio L. F. Natalicio D. S. (1971). The state-trait anxiety inventory. *Int. J. Psychol.* 5 145–158.

[B27] Strong M. J. Abrahams S. Goldstein L. H. Woolley S. Mclaughlin P. Snowden J. (2017). Amyotrophic lateral sclerosis-frontotemporal spectrum disorder (ALS-FTSD): revised diagnostic criteria. *Amyotrophic Lateral Sclerosis Frontotemporal Degenerat.* 18 153–174. 10.1080/21678421.2016.1267768 28054827 PMC7409990

[B28] Tremolizzo L. Lizio A. Santangelo G. Diamanti S. Lunetta C. Gerardi F. (2020). ALS Cognitive Behavioral Screen (ALS-CBS): normative values for the Italian population and clinical usability. *Neurol. Sci.* 41 835–841. 10.1007/s10072-019-04154-1 31807998

[B29] Woolley S. Goetz R. Factor-Litvak P. Murphy J. Hupf J. Lomen-Hoerth C. (2018). Longitudinal screening detects cognitive stability and behavioral deterioration in ALS patients. *Behav. Neurol.* 2018:5969137. 10.1155/2018/5969137 30515252 PMC6234441

[B30] Woolley S. C. York M. K. Moore D. H. Strutt A. M. Murphy J. Schulz P. E. (2010). Detecting frontotemporal dysfunction in ALS: utility of the ALS Cognitive Behavioral Screen (ALS-CBS™). *Amyotrophic Lateral Sclerosis* 11 303–311. 10.3109/17482961003727954 20433413

